# The clinical usefulness of guideline-based strategies with and without the role of nonspecific symptoms to predict urinary tract infections in nursing homes: a decision curve analysis

**DOI:** 10.1017/ash.2024.345

**Published:** 2024-08-01

**Authors:** Sacha Kuil, Menno de Jong, Caroline Schneeberger, Frank van Leth

**Affiliations:** 1 Amsterdam UMC, University of Amsterdam, Department of Medical Microbiology, Amsterdam Infection & Immunity Institute, Amsterdam, The Netherlands; 2 Department of Health Sciences, Vrije Universiteit, Amsterdam, The Netherlands

## Abstract

**Objective:**

The aim of this study was to assess the clinical value of urinary tract infections (UTIs) guideline algorithms and the role of nonspecific symptoms to support clinical decision-making in nursing home residents.

**Design:**

In a preplanned secondary analysis of a cross-sectional study including nursing home residents with a presumed UTI, 2 prediction models were used in a decision curve analysis (DCA): (1) guideline-based and (2) extended: nonspecific symptom(s) added to the guideline model. The stringent outcome definition for “true UTIs” included symptom improvement during adequate antimicrobial therapy, based on susceptibility test results. The outcome of a DCA is the Net Benefit to quantify the performance of the prediction models, visualized in a decision curve.

**Setting:**

Dutch nursing homes (n = 13).

**Patients:**

Nursing home residents with a presumed UTI.

**Results:**

Of the 180 residents with a presumed UTI, 43 fulfilled the definition of “true UTI” (23.9%). The Net Benefit of the guideline-based model was low and the corresponding threshold range was small (21%–28%). The extended model improved the prediction of UTIs. However, the clinical usefulness of the extended model was still limited to a small threshold range (10%–28%).

**Conclusions:**

The clinical usefulness of the current guideline-based algorithm to diagnose UTI in nursing home residents seems limited, and adding nonspecific symptoms does not further improve decision-making due to the small threshold probability. Given the poor performance of the guideline-based model, refinement of the guidelines may be required.

**Trial registry:**

Dutch trial registry: NTR6467; date of first registration, 25/05/2017.

## Introduction

Diagnosing a urinary tract infection (UTI) in nursing home residents is difficult. This is due to a variety of factors, including the lack of appropriate diagnostic tests for UTIs to differentiate them from asymptomatic bacteriuria (ASB), which is highly prevalent in this population.^1^ Expression of specific urinary signs and symptoms due to cognitive impairments is often difficult for residents,^2^ and from the side of caregivers, assessment of these symptoms is complex.^3^ Nonspecific symptoms, such as confusion, are highly prevalent and historically attributed to UTIs.^3,4^

The role of nonspecific symptoms is unclear in nursing home residents suffering from UTI. The latest international guidelines and criteria (such as the Loeb criteria), although created for surveillance purposes, do not consider nonspecific symptoms in the diagnosis of UTI in older adults. ^5–11^ The recommendations are based on inconsistent results on the role of nonspecific symptoms and UTI,^9,12–19^ including 3 meta-analysis providing conflicting evidence.

To our knowledge, the diagnostic value of the guideline-based criteria for UTI in older adults upon implementation has not been described. Several prediction models for UTI diagnosis in other populations, for example, children and women, or settings such as emergency departments have been developed,^20–23^ as well as a model to predict adverse outcomes in geriatric patients with UTI.^24^ Caterino and colleagues^18^ developed a model to predict whether an altered mental status and malaise/lethargy affect the posttest probability of infection in older adults admitted to the emergency department. The probability of infections overall did not increase, but the probability of a UTI increased moderately when using their model.

The objective of this study was to assess the clinical relevance of the implemented guideline-based diagnostic criteria for UTI and the added value of nonspecific symptoms to support clinical decision-making.

## Methods

### Design

This was a preplanned secondary analysis of a cross-sectional study (PROGRESS) with the overall aim to improve UTI diagnosis in nursing homes. A full description of the study design and procedures has been described elsewhere.^25^

### Setting and participants

The data were collected between November 2017 and August 2019 in 13 Dutch nursing homes. The intention was to resume the study after an initial interim analysis-related break for the primary study objective. At the beginning of 2020, we decided to end the study permanently due to the closing of the nursing homes in the coronavirus disease 2019 (COVID-19) pandemic. Consenting older adults (≥65 years old) residing at a psychogeriatric, somatic, or rehabilitation ward with a presumed UTI based on clinical assessment of the attending clinician were eligible for enrollment. Exclusion criteria were (1) presumed respiratory tract infection (RTI) or other infection requiring antibiotics, (2) previous enrollment, or (3) urine collected >24 hours after initiation of antibiotic therapy. In Dutch nursing homes, elderly care physicians (physicians who specialize in long-term care for frail elderly people for 3 years) are responsible for the medical care of residents. Specially trained nurses can perform delegated tasks such as clinical assessment and diagnostic decisions.

### Reference test for UTI

As there is no reference test for UTI diagnosis in this population, a stringent composite definition for “true UTI” was used. To fulfill this definition, one should meet all of the following criteria: (1) a positive urine leucocyte esterase test, (2) uropathogens in a bacterial culture at ≥104 colony-forming units/mL; (3) a maximum of 2 uropathogens; and (4) improvement of symptoms during follow-up while adequate antibiotic treatment was received (ie, proven susceptibility of isolated uropathogens to the administered antibiotic). The type of symptoms was not part of the “true UTI” definition. Treating physicians decided whether or not to treat based on their own clinical decision-making, without having access to the results of study procedures. As such, the clinical practice in the nursing home was not altered by the study activities. All primary and secondary uropathogens in the European Consensus Guideline were considered as uropathogens.^26^ Other bacterial species were considered as non-uropathogens. Whether the initial symptoms improved was reported at day 5 and 10 during follow-up. The improvement of symptoms at day 10 was used to determine the presence of a “true UTI.” When data at day 10 was missing or reported to be unknown, symptom improvement at day 5 was used. When the improvement was reported as unknown at day 5, this was considered to be no improvement.

### Data collection

The presence of specific urinary symptoms (dysuria, frequency, urgency, new or worsened urine incontinence, urethral purulence, lower abdominal pain, costovertebral angle tenderness, and hematuria), systemic signs and symptoms (fever, chills, delirium), nonspecific symptoms (confusion, malaise, and loss of appetite), and an indwelling urinary catheter were reported on a clinical report form (CRF) by the attending clinician at the time of suspicion of UTI (day 0). When the presence of symptoms was unknown, this was considered to be an absence of symptoms. The procedures for urine collection, urine dipstick tests, and bacterial cultures were described previously.^25^ Participants and attending clinicians were not informed about urine dipstick and bacterial culture results. Symptom improvement was assessed and registered on the CRF at day 5 and 10 of follow-up.

### Outcome and analysis

We used a decision curve analysis as proposed by Vickers and Elkin.^27^ The Net Benefit is the outcome measure of a decision curve analysis to quantify the performance of different prediction models. The Net Benefit denotes the difference between the true positive and false positive classifications of the outcome (UTI) based on the prediction model and is calculated by the following formula: (TP – (w * FP))/N, where TP = true positives, w = weight (threshold probability), FP = false positives, and N = total number of individuals. The threshold probability in this formula is defined as the minimum probability of disease above which the physician considers a UTI to be present and decides to treat. The treating physician chooses a reasonable threshold probability that reflects clinical considerations on the risk of under- and overtreatment.^28^ Personal preferences and attitudes of the prescriber may also play a role.^29^ Diagnostic and treatment thresholds of 19.1% and 42.3%, respectively, were described previously in suspected UTI in primary care. ^30^ The Net Benefit of the prediction models is compared with “treating all” or “treating none” of the individuals. An elderly care physician might only need a low clinical suspicion for UTI to treat a patient with the idea that antibiotics might improve the presenting symptoms and prevent the worsening toward pyelonephritis. On the other hand, antibiotics may lead to side effects such as *Clostridioides difficile* infection, drug-drug interactions, or antimicrobial resistance development. These considerations might result in elderly care physicians to require a higher clinical suspicion of UTI before starting treatment. Decision curve analysis incorporates these different considerations in thresholds probabilities for treatment. It translates the minimal pretest probability for the outcome (UTI) at which the physician is willing to start treatment into the clinical threshold with different weights for false positives (risk for adverse events and antimicrobial resistance) and false negatives (risk for persisting or worsening of symptoms) of the prediction model. When a threshold probability of 10% is chosen, the harm of delaying antimicrobial treatment is considered 9 times higher than the harm of unnecessarily treating with antibiotics for that particular patient (10% reflects a ratio 1:9).

We compared 2 different models. The first is the guideline-based model, in which UTI is assumed to be present if there is at least 1 specific urinary symptom, together with a positive leukocyte or nitrite dipstick results.^5,9^ This model reflects the prevailing guideline in the Netherlands. The second is the extended model, in which a UTI is assumed if the criteria of the guideline model are met, together with at least 1 nonspecific symptom.

The Net Benefit of the 2 prediction models was visualized as a function of the threshold probability in a decision curve. We calculated the Net Benefit for a range of thresholds (0%–40%), as thresholds are known to vary between type of practitioners and number of years in practice.^30^ A model is clinically useful when the Net Benefit is higher than the Net Benefit of the models that reflect to treat all or treat none of the patients.

The assessment of predictors was blinded to the outcome measure, as we classified for the outcome (true UTI) after data collection, because symptom improvement was part of our stringent definition. No prestudy sample size calculations were performed. All enrollments were used for analysis; there were no possibilities to extend the study due to COVID-19. No repeated enrollments were used for analysis to ensure independence of the observations. For the reference true UTI definition, complete case analysis was used, and enrollments with missing test results were excluded.

Calculations and decision curves were performed in RStudio (dca function) version 1.4.1103 (Integrated Development for R. RStudio, Inc., Boston, MA).

The reporting of the study is in accordance with the TRIPOD guideline (transparent reporting of a multivariate prediction model for individual prognosis or diagnosis).^31^

## Results

### Participants

**Figure 1.**
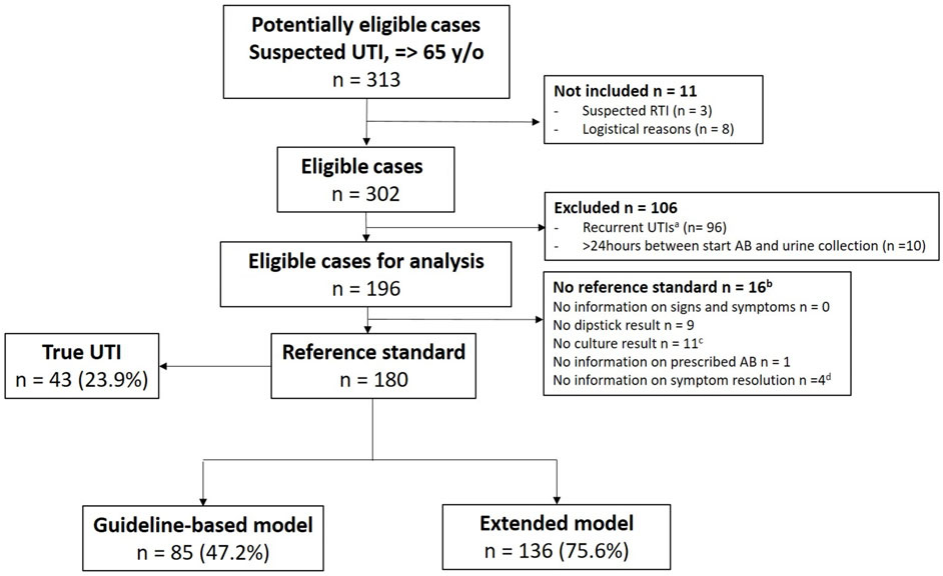
Flow of eligible UTI cases, number of “true UTIs,” and number of UTIs based on the guideline (n = 85) and extended model (n = 136). AB, antibiotics; RTI, respiratory tract infection; UTI, urinary tract infection. ^a^ Single UTIs were included, and of the recurrent cases, a random case for each individual was used. ^b^ Multiple missing items required for reference testing can occur within 1 UTI case. ^c^ No urine culture result or no info about urine collection method. ^d^ No information about symptom improvement at day 5 and day 10.

The study enrolled 313 presumed UTI cases. After exclusions, there were 196 eligible presumed UTI cases of which 180 had all mandatory items documented for the stringent “true UTI” definition that represented the study population (Figure 1).

### Characteristics of participants

Most participants were female (78.9%), and the median age was 87 years (range 66–107). Participants enrolled stayed at psychogeriatric, rehabilitation, and somatic wards (72.8%, 13.9%, and 13.3%, respectively) which is in line with the composition of nursing home wards in the Netherlands. The frequency of reported specific and nonspecific symptoms are listed in Supplementary Table S1–S2.

### Frequency of “true UTIs”

Of the 180 presumed UTI cases, the urine leukocyte esterase and/or nitrite test was positive in 151 cases (83.9%; Table 1). In 71 of 151 cases, bacterial cultures were positive according to our definition (47.0%), and 59 cases were treated with adequate antibiotics according to the susceptibility test results (83.1%, 59/71). The most common uropathogens in this study were *Escherichia coli* (62%), *Proteus* spp. (8.9%), and *Aerococcus* spp. (8.3%). In 43 cases, the initial symptoms improved during antibiotic therapy, fulfilling the definition of “true UTI” (23.9%). Most UTIs were treated with nitrofurantoin (64.0%) and amoxicillin-clavulanic acid (19.3%).

**Table 1.** Presence of the criteria to fulfill the composite outcome UTI

**Dipstick results** ^a^		
Leucocytes and/or nitrite positive	151	83.9% (151/180)
**Bacterial culture results** ^b^		
Number of positive bacterial cultures	71	47.0% (71/151)^c^
**Antibiotic therapy covering uropathogen(s)** ^d^		
Number of prescribed antibiotics covering identified uropathogen(s)	59	83.1% (59/71)^e^
**Symptom improvement** ^f^		
Symptom improvement	43	72.9% (43/59)
**Total number of “true” UTI**		
UTI present based on composite outcome measure	43	23.9% (43/180)

Note. UTI, urinary tract infection.

a Positive urine leucocyte esterase test: ≥1+ leukocytes detected by Combur2 dipstick analysis.

b Positive bacterial culture defined as the presence of 1 or 2 uropathogens at ≥104 CFU/mL.

c Most bacterial cultures (23) were negative among the dipstick-negative urine specimens (79.3%).

d Proven susceptibility of isolated uropathogens to the administered antibiotic, in case of positive urine culture.

e The symptoms improved in 9 of the 12 residents treated with an antibiotic not covering the identified uropathogen.

f Improvement of the initially presented symptoms during follow-up; unknown symptom improvement equals the absence of improvement; in case of missing data on symptom improvement at day 10, symptom improvement at day 5 was used.

### Primary outcome: the Net Benefit for UTI prediction

**Figure 2.**
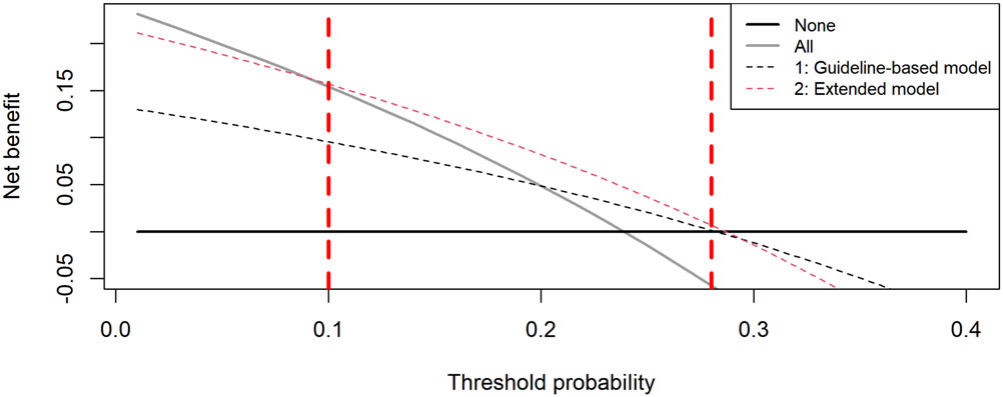
Results of the decision curve analysis. Net Benefit (y-axis) for the prediction of UTI as a function of the threshold probability (x-axis), for (1) the guideline-based model: inclusion of only specific urinary symptoms and positive leukocyte or nitrite dipstick results (black dotted line); (2) the extended model: according to current guideline or ≥1 nonspecific symptoms with positive leukocyte or nitrite dipstick test, (red dotted line), compared with the treating all patients (gray line) and treating none (black line). The Net Benefit for the extended model including nonspecific symptoms was higher compared with the guideline-based model, treat all and treat none over the range of 10%–28% (red dashed vertical lines). Treating all patients is the best strategy below the threshold of 10%, and treating none is the best strategy above the threshold of 28% (red dashed vertical lines). UTI, urinary tract infection.

The guideline-based model showed a higher Net Benefit compared with “treating all” or “treating none,” indicating clinical usefulness (Figure 2). However, this model lacked clinical usefulness for the threshold probabilities below 21% and above 28%.

The extended model showed a higher Net Benefit and a wider corresponding threshold probability range (10%–28%) compared with the guideline-based model. The extended model was clinically useful for physicians who consider missing a UTI to be about 2.5 to 9 times worse than unnecessarily treating with antibiotics. Physicians who consider lower (<10%) or higher (>28%) thresholds for treatment (very worried about UTI or very worried about adverse events, respectively) should avoid using the extended model and rather treat all individuals or no individuals with a suspected UTI, respectively. The Net Benefit of the extended model ranged from 0.07 (at a risk threshold of 28%) to 0.157 (at a risk threshold of 10%). The unit of the Net Benefit is the net true positives.^32^ This equates to the treatment of 7–15.7 patients with a true UTI per 100 patients suspected for having a UTI by the extended model, with no patients unnecessarily treated with antibiotics.

**Figure 3.**
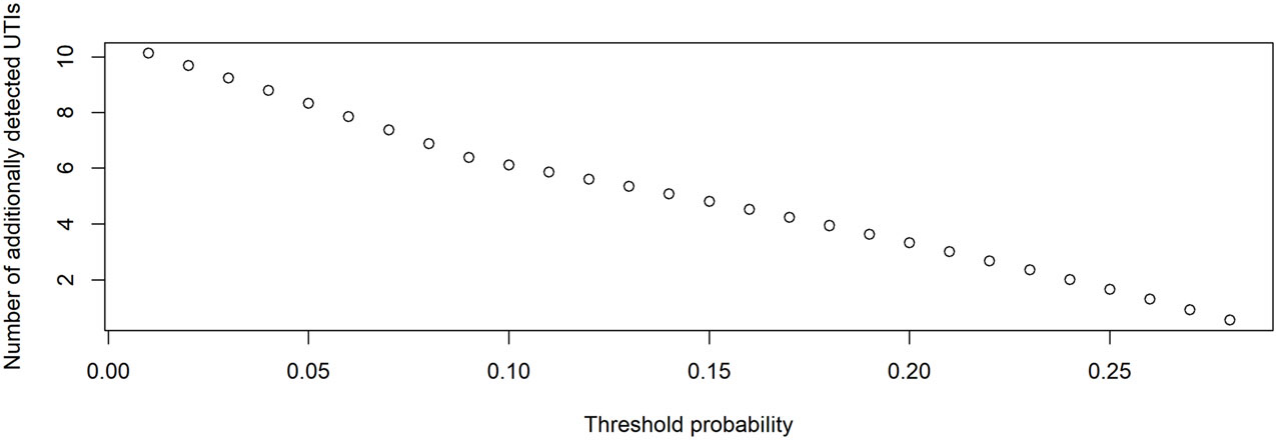
Results of the decision curve analysis. Plot demonstrating the additional detected UTIs without unnecessary antibiotic treatments if the extended model is used rather than the guideline-based model per 100 patients with a presumed UTI, as a function of the risk threshold range 0%–28%. UTI, urinary tract infection.

When comparing the extended model and the guideline-based model, the additionally detected true UTIs ranged from 0.6 to 10.1 per 100 patients with a suspected UTI (Figure 3).

To illustrate the translation of the guideline-based model compared with the extended model into clinical usefulness, we use an example. Let us assume that an elderly care physician considers missing a UTI to be 4 times worse than unnecessarily treating a particular patient with antibiotics. This 4:1 ratio is equal to a preferred threshold probability of 20%. In this case, the Net Benefits of the guideline-based model and the extended model are 0.0486 and 0.0819, respectively (Table 2). This means that the difference in Net Benefit between the guideline-based and the extended model is 0.0333, which translates into 3.33 additionally detected UTIs per 100 patients for whom antibiotic treatment is appropriate when the extended model is used (Table 2). The extended model improved the prediction of a UTI, compared with the guideline-based model, but the threshold probability range was narrow.

**Table 2.** Net Benefits for the guideline-based and extended model to predict urinary tract infections (UTI) at a probability threshold of 20%

Statistic	Result
**Default strategy**	
Net Benefit for treat all	0.0486
Net Benefit for treat none	0
**Guideline-based model**	
Net Benefit if the guideline-based model was used to select for treatment	0.0486
Detected UTIs without unnecessary antibiotic treatments	4.9 per 100 patients
**Extended versus guideline-based model**	
Net Benefit if the extended model is used to select for treatment	0.0819
Net Benefit difference between guideline-based and extended model	0.0333
Additional detected UTIs without unnecessary antibiotic treatments if the extended model is used rather than the guideline-based model	3.33 per 100 patients

Net Benefit = (TP – (w * FP))/N, where TP = true positives, w = weight (threshold probability), FP = false positives, and N = total number of patients. Guideline-based model: specific urinary symptoms and positive urine dipstick result; extended model: guideline-based model or ≥1 nonspecific symptoms with positive dipstick results.

## Discussion

The clinical usefulness of diagnosing UTI based on the current guideline algorithms is limited. Our extended model which adds nonspecific symptoms to the guideline algorithm improved the prediction of UTI; however, even this extended model is clinically useful for only a small range of physicians’ preferred threshold probabilities. Outside these thresholds, treating all or no patients with a presumed UTI is preferred.

The results of this study indicate that there is only a small window of opportunity to improve decision-making by using nonspecific symptoms. Whether this small threshold probability range is clinically useful depends on the particular physicians’ preferences, which implicitly incorporates the anticipated treatment effect (the more effective the treatment for health benefits, the lower the threshold) and the estimated risks of harm by under- and overtreatment for the specific patient (individualized medicine). Threshold probability ranges are by definition subjective and vary between physicians.^32^ The number of false positives receiving unnecessary antibiotics is high using both models (Supplementary Tables 4 and 5). The harms of unnecessary antimicrobial use in terms of adverse events might be acceptable at the individual level, but the harm of antimicrobial resistance development has consequences at local or global population levels, and whether resistance development affects individual treatment decisions is unsure.^33,34^ Although based on our results no direct recommendations to incorporate nonspecific symptoms to treatment algorithms can be made, the observed limited usefulness of the guideline-based model based on specific symptoms and positive dipstick results is noteworthy. This finding is supported by previous meta-analysis illustrating a limited diagnostic value of specific urinary symptoms in older adults.^35^ Furthermore, a decrease in diagnostic value of specific urinary symptoms with increasing age has been reported by others.^36^ The limiting factor of these 2 studies is that they use bacterial cultures as a reference test for UTI which is less informative in a setting with a high prevalence of asymptomatic bacteriuria. We tried to minimize this limitation by adding treatment response in our UTI definition. When using a very stringent UTI definition, one may expect a high number of true positives and a low number of false negatives. In our study, only 24 of the 43 “true UTIs” were detected using the guideline-based model, compared with 39 UTIs when using the extended model (Supplementary Tables 4 and 5). This illustrates that using solely specific urinary symptoms and positive dipstick results may contribute to the underdiagnosis of UTIs in nursing home residents. The guideline model to predict UTI thus seems barely clinically useful, and because appropriate diagnostic tests to differentiate UTI from ASB are currently lacking, physicians need their clinical expertise to make a judgment call with regard to treatment.

Decision curve analyses have not previously been described for the evaluation of diagnostic strategies for UTI in this population. Decision analysis is a research tool and is not designed to be used for individual residents. It is a method to try to determine the value of a prediction model in clinical practice. We used the guideline-based model without assessing its discriminatory power, as the model is already fully implemented in Dutch nursing homes. The same holds true for the extended model as it adds just a single criterion to the implemented guideline-based model.

It is known that assessment of clinical symptoms varies among physicians,^37^ that nonspecific symptoms can cause diagnostic uncertainty, and that a diagnostic test to support decision-making is preferred.^38^ Our findings that show the limited usefulness of symptomatology further underline the need for appropriate diagnostic tests to distinguish patients with a UTI from ASB.

The added value of this study to the existing literature is the use of an analysis approach to assess the clinical usefulness of a model by weighing true positives and false positives, rather than traditional measures for discrimination, as treatment decisions are likely to vary among physicians. Using traditional measures like receiver operating curves with false positives and false negatives equally weighted results in more weight for positive outcomes when a positive outcome is less common (as in our study).^39^ This study illustrates the potential usefulness of the decision curve methodology as an alternative solution in further studies to define the best approach to the diagnosis of UTI in nursing home residents.

A limitation of our study was the very few events of UTI. Second, we used a stringent definition for “true UTIs” consisting of 4 items, including symptom resolution during adequate antibiotic treatment, to reduce misclassification of the outcome. This stringent definition may have underestimated the true number of UTIs because treatment response was part of the definition and not every patient was treated. On the other hand, the number of true UTIs may have been overestimated when symptoms unrelated to UTI resolved spontaneously. We deliberately included the therapy effect because it leads to the least overestimation of the UTIs. By leaving the treatment decision entirely to the physician, the results can be generalized to a large extent. Without a reference test available for UTI, we consider using this stringent definition as a reasonable solution. For future studies, a research reference standard could be used for which efforts are already being made to develop one.^40^ Third, the presence of symptoms used in the prediction models was based on the clinical assessment of attending clinicians rather than using standardized assessment scales,^41^ but we assumed this reflected the clinical practice. Finally, the criterion for inclusion was a presumed UTI based on clinical assessment, which may have varied between physicians or with time due to the publication of the latest UTI guideline in October 2018. This may have caused clinical heterogeneity, affecting the pretest probability of having a UTI.

## Conclusions and implications

The current guideline-based diagnostic algorithm seems to have a limited clinical usefulness in the prediction of UTI requiring antibiotic treatment. Using nonspecific symptoms might improve the prediction of UTI to a limited extent. Our findings imply that symptomatology offers little guidance for clinicians, that current guidelines focusing on specific urinary symptoms may need to be nuanced, and that there is a need for a reliable diagnostic tool to guide appropriate antibiotic treatment for UTIs in the geriatric population.

## Supporting information

Kuil et al. supplementary material Kuil et al. supplementary material

## Data Availability

The data sets are available on a dedicated project page: doi: 10.17605/OSF.IO/GDR8F.

## Supplementary material

For supplementary material accompanying this paper visit http://doi.org/10.1017/ash.2024.345. click here to view supplementary material

## References

[1] Nicolle LE , Gupta K , Bradley SF , et al. Clinical practice guideline for the management of asymptomatic bacteriuria: 2019 update by the infectious diseases society of America. Clin Infect Dis 2019;68:e83–e110. 30895288 10.1093/cid/ciy1121

[2] Nicolle LE , Strausbaugh LJ , Garibaldi RA. Infections and antibiotic resistance in nursing homes. Clin Microbiol Rev 1996;9:1–17. 8665472 10.1128/cmr.9.1.1 PMC172878

[3] D’Agata E , Loeb MB , Mitchell SL. Challenges in assessing nursing home residents with advanced dementia for suspected urinary tract infections. J Am Geriatr Soc 2013;61:62–66. 23311553 10.1111/jgs.12070 PMC3545416

[4] Arinzon Z , Shabat S , Peisakh A , Berner Y. Clinical presentation of urinary tract infection (UTI) differs with aging in women. Arch Gerontol Geriatr 2012;55:145–147. 21963175 10.1016/j.archger.2011.07.012

[5] Hertogh C , Haaijman J. Richtlijn Urineweginfecties bij kwetsbare ouderen. *Verenso* 2018.

[6] McGeer A , Campbell B , Emori TG et al. Definitions of infection for surveillance in long-term care facilities. Am J Infect Control 1991;19:1–7. 1902352 10.1016/0196-6553(91)90154-5

[7] Stone ND , Ashraf MS , Calder J , et al. Surveillance definitions of infections in long-term care facilities: revisiting the McGeer criteria. Infect Control Hosp Epidemiol 2012;33:965–977. 22961014 10.1086/667743 PMC3538836

[8] al. NLe. Infectious Diseases Society of America guidelines for the diagnosis and treatment of asymptomatic bacteriuria in adults. Clinical Infectious Diseases 2005;40:643–654. 15714408 10.1086/427507

[9] van Buul LW , Vreeken HL , Bradley SF , et al. The development of a decision tool for the empiric treatment of suspected urinary tract infection in frail older adults: a Delphi consensus procedure. Journal of the American Medical Directors Association 2018;19:757–764. 29910137 10.1016/j.jamda.2018.05.001 PMC8048094

[10] (SIGN) SIGN. Management of suspected bacterial urinary tract infection in adults. 2012. https://www.sign.ac.uk/media/1051/sign88.pdf. Accessed 21 May 2021.

[11] Loeb M , Bentley DW , Bradley S , et al. Development of minimum criteria for the initiation of antibiotics in residents of long-term-care facilities: results of a consensus conference. Infect Control Hosp Epidemiol 2001;22: 120–124. 11232875 10.1086/501875

[12] Sundvall PD , Ulleryd P , Gunnarsson RK. Urine culture doubtful in determining etiology of diffuse symptoms among elderly individuals: a cross-sectional study of 32 nursing homes. BMC Fam Pract 2011;12:36. 21592413 10.1186/1471-2296-12-36 PMC3142216

[13] Ducharme J , Neilson S , Ginn JL. Can urine cultures and reagent test strips be used to diagnose urinary tract infection in elderly emergency department patients without focal urinary symptoms? Cjem 2007;9:87–92. 17391578 10.1017/s1481803500014846

[14] Balogun SA , Philbrick JT. Delirium, a symptom of UTI in the elderly: fact or fable? A systematic review. Can Geriatr J 2014;17:22–26. 24596591 10.5770/cgj.17.90 PMC3940475

[15] Mayne S , Bowden A , Sundvall PD , Gunnarsson R. The scientific evidence for a potential link between confusion and urinary tract infection in the elderly is still confusing - a systematic literature review. BMC Geriatr 2019;19:32. 30717706 10.1186/s12877-019-1049-7 PMC6360770

[16] Krinitski D , Kasina R , Klöppel S , Lenouvel E. Associations of delirium with urinary tract infections and asymptomatic bacteriuria in adults aged 65 and older: a systematic review and meta-analysis. J Am Geriatr Soc 2021;69:3312–3323. 34448496 10.1111/jgs.17418 PMC9292354

[17] Penna AR , Sancken CL , Stone ND , et al. Documentation of acute change in mental status in nursing homes highlights opportunity to augment infection surveillance criteria. Infect Control Hosp Epidemiol 2020;41:848–850. 32340639 10.1017/ice.2020.77 PMC8668157

[18] Caterino JM , Kline DM , Leininger R , et al. Nonspecific symptoms lack diagnostic accuracy for infection in older patients in the emergency department. J Am Geriatr Soc 2019;67:484–492. 30467825 10.1111/jgs.15679 PMC6403002

[19] Boscia JA , Kobasa WD , Abrutyn E , Levison ME , Kaplan AM , Kaye D. Lack of association between bacteriuria and symptoms in the elderly. Am J Med 1986;81:979–982. 3799658 10.1016/0002-9343(86)90391-8

[20] Shaikh N , Hoberman A , Hum SW , et al. Development and validation of a calculator for estimating the probability of urinary tract infection in young febrile children. JAMA Pediatr 2018;172:550–556. 29710324 10.1001/jamapediatrics.2018.0217 PMC6137527

[21] Taylor RA , Moore CL , Cheung KH , Brandt C. Predicting urinary tract infections in the emergency department with machine learning. PLoS One 2018;13:e0194085. 29513742 10.1371/journal.pone.0194085 PMC5841824

[22] Foudraine DE , Bauer MP , Russcher A , et al. Use of automated urine microscopy analysis in clinical diagnosis of urinary tract infection: defining an optimal diagnostic score in an academic medical center population. J Clin Microbiol 2018;56:e02030-17. 10.1128/JCM.02030-17 PMC5971551 29643200

[23] Gágyor I , Haasenritter J , Bleidorn J , McIsaac W , Schmiemann G , Hummers-Pradier E , Himmel W. Predicting antibiotic prescription after symptomatic treatment for urinary tract infection: development of a model using data from an RCT in general practice. Br J Gen Pract 2016;66:e234–240. 26965031 10.3399/bjgp16X684361 PMC4809706

[24] Ginde AA , Rhee SH , Katz ED. Predictors of outcome in geriatric patients with urinary tract infections. J Emerg Med 2004;27:101–108. 15261349 10.1016/j.jemermed.2004.02.015

[25] Kuil SD , Hidad S , Fischer JC , et al. Sensitivity of point-of-care testing C reactive protein and procalcitonin to diagnose urinary tract infections in Dutch nursing homes: PROGRESS study protocol. BMJ Open 2019;9:e031269. 10.1136/bmjopen-2019-031269 PMC6701568 31401614

[26] Aspevall O , Hallander H , Gant V , Kouri T. European guidelines for urinalysis: a collaborative document produced by European clinical microbiologists and clinical chemists under ECLM in collaboration with ESCMID. Clin Microbiol Infect 2001;7:173–178. 11422238 10.1046/j.1198-743x.2001.00237.x

[27] Vickers AJ , Elkin EB. Decision curve analysis: a novel method for evaluating prediction models. Med Decis Making 2006;26:565–574. 17099194 10.1177/0272989X06295361 PMC2577036

[28] Vickers AJ , van Calster B , Steyerberg EW. A simple, step-by-step guide to interpreting decision curve analysis. Diagn Progn Res 2019;3:18. 31592444 10.1186/s41512-019-0064-7 PMC6777022

[29] Ebell MH , Locatelli I , Mueller Y , Senn N , Morgan K. Diagnosis and treatment of community-acquired pneumonia in patients with acute cough: a quantitative study of decision thresholds in primary care. Br J Gen Pract 2018;68:e765–e774. 30348882 10.3399/bjgp18X699545 PMC6193794

[30] Harris A , Pineles L , Baghdadiv JD , et al. Clinician testing and treatment thresholds for management of urinary tract infection. Open Forum Infect Dis 2023;10:ofad455. 37720701 10.1093/ofid/ofad455 PMC10500043

[31] Collins GS , Reitsma JB , Altman DG , Moons KG. Transparent Reporting of a multivariable prediction model for Individual Prognosis or Diagnosis (TRIPOD): the TRIPOD statement. Ann Intern Med 2015;162:55–63. 25560714 10.7326/M14-0697

[32] Van Calster B , Wynants L , Verbeek JFM , et al. Reporting and interpreting decision curve analysis: a guide for investigators. Eur Urol 2018;74:796–804. 30241973 10.1016/j.eururo.2018.08.038 PMC6261531

[33] Nicholson A , Tennant I , White L , et al. Correction to: the knowledge, attitudes and practices of doctors regarding antibiotic resistance at a tertiary care institution in the Caribbean. Antimicrob Resist Infect Control 2018;7:77. 29983930 10.1186/s13756-018-0358-5 PMC6019706

[34] Zhuo A , Labbate M , Norris JM , et al. Opportunities and challenges to improving antibiotic prescribing practices through a One Health approach: results of a comparative survey of doctors, dentists and veterinarians in Australia. BMJ Open 2018;8:e020439. 10.1136/bmjopen-2017-020439 PMC5884343 29602857

[35] Gbinigie OA , Ordóñez-Mena JM , Fanshawe TR , Plüddemann A , Heneghan C. Diagnostic value of symptoms and signs for identifying urinary tract infection in older adult outpatients: systematic review and meta-analysis. J Infect 2018;77:379–390. 29964141 10.1016/j.jinf.2018.06.012 PMC6203890

[36] Holm A , Siersma V , Cordoba GC. Diagnosis of urinary tract infection based on symptoms: how are likelihood ratios affected by age? a diagnostic accuracy study. BMJ Open 2021;11:e039871. 10.1136/bmjopen-2020-039871 PMC7798711 33419902

[37] Hughes C , Ellard DR , Campbell A , et al. Developing evidence-based guidance for assessment of suspected infections in care home residents. BMC Geriatr 2020;20:59. 32059649 10.1186/s12877-020-1467-6 PMC7023778

[38] Kuil SD , Schneeberger C , van Leth F , de Jong MD , Harting J. “A false sense of confidence” The perceived role of inflammatory point-of-care testing in managing urinary tract infections in Dutch nursing homes: a qualitative study. BMC Geriatr 2020;20:450. 33148189 10.1186/s12877-020-01853-9 PMC7643302

[39] Steyerberg EW , Vickers AJ , Cook NR , et al. Assessing the performance of prediction models: a framework for traditional and novel measures. Epidemiology 2010;21:128–138. 20010215 10.1097/EDE.0b013e3181c30fb2 PMC3575184

[40] M. B. UTI Reference Standard: Delphi Method (ORACLE). 2022. https://clinicaltrials.gov/ct2/show/NCT05365906.

[41] Wei LA , Fearing MA , Sternberg EJ , Inouye SK. The confusion assessment method: a systematic review of current usage. J Am Geriatr Soc 2008;56:823–830. 18384586 10.1111/j.1532-5415.2008.01674.x PMC2585541

