# Supplementary material for: The clinical usefulness of guideline-based strategies with and without the role of nonspecific symptoms to predict urinary tract infections in nursing homes: a decision curve analysis
Source: Antimicrob Steward Healthc Epidemiol. 2024 Aug 1;4(1):e105. doi: 10.1017/ash.2024.345 (PMC11588415; doi:10.1017/ash.2024.345)
Supplement: Kuil et al. supplementary material [file S2732494X24003450sup001.docx]

**Supplementary Table S1-S5**

**Supplementary table S1 Symptoms reported by the treating clinician**

| **Specific urinary symptoms** | |
| --- | --- |
| Abdominal pain | 57 (31.7%) |
| Dysuria | 40 (22.2%) |
| Frequency | 69 (38.3%) |
| Purulent discharge | 5 (2.8%) |
| Urgency | 77 (42.8%) |
| Urinary incontinence (worsened) | 33 (18.3%) |
| **Non-specific symptoms** | |
| Confusion | 96 (53.3%) |
| Malaise | 61 (33.9%) |
| Loss of appetite | 37 (20.6%) |
| **Systemic symptoms** | |
| Fever | 10 (5.6%) |
| Delirium | 20 (11.1%) |
| Shivers | 7 (3.9%) |
| Costovertebral angle tenderness | 8 (4.4%) |
| **Presence of indwelling catheter** | |
| **Indwelling catheter** | 9 (5.0%) |

**Supplementary table S2 Number of symptoms reported by the treating clinician per resident**

| ***Number of specific symptoms^1^ reported per resident*** | |
| --- | --- |
| *0* | *52 (28.9%)* |
| *1* | *45 (25.0%)* |
| *2* | *37 (20.6%)* |
| *3* | *26 (14.4%)* |
| *4* | *16 (8.9%)* |
| *5* | *4 (2.2%)* |
| *6* | *0 (0%)* |
| ***Number of systemic symptoms^2^ reported per resident*** | |
| *0* | *147 (81.7%)* |
| *1* | *29 (16.1%)* |
| *2* | *4 (2.2%)* |
| *3* | *0 (0%)* |
| ***Number of nonspecific symptoms^3^ reported per resident*** | |
| *0* | *54 (30.0%)* |
| *1* | *71 (39.4%)* |
| *2* | *42 (23.3%)* |
| *3* | *13 (7.2%)* |

*^1^ dysuria, frequency, urgency, worsened urine incontinence, purulent discharge, abdominal pain ^2^ fever, chills, delirium ^3^ confusion, malaise, loss of appetite*

**Supplementary Figure S3 Symptom distribution among UTI episodes**


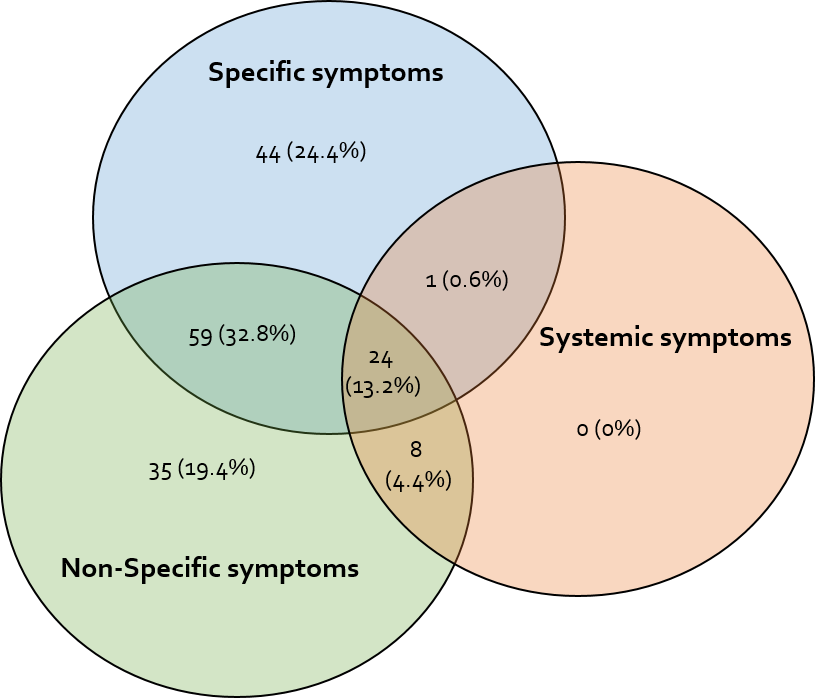


**Supplementary table S4 2x2 table Model 1 Guideline-based**

|  |  |  | **Model 1: Guideline-based** | | |
| --- | --- | --- | --- | --- | --- |
|  |  |  | UTI absent (0) | UTI present (1) | **Total** |
| **UTI outcome** | UTI absent (0) | Counts | 76 | 61 | 137 |
|  |  | Expected | 72.3 | 64.7 |  |
|  |  | Residuals | 3.7 | -3.7 |  |
|  | UTI present (1) | Counts | 19 | 24 | 43 |
|  |  | Expected | 22.7 | 20.3 |  |
|  |  | Residuals | -3.7 | 3.7 |  |
|  | **Total** | | **95** | **85** | **180** |

TP = 24, FP = 61

**Supplementary table S5 2x2 table Model 2: Extended model**

|  |  |  | **Model 2:Extended model** | | |
| --- | --- | --- | --- | --- | --- |
|  |  |  | UTI absent (0) | UTI present (1) | **Total** |
| **UTI outcome** | UTI absent (0) | Counts | 40 | 96 | 137 |
|  |  | Expected | 33.5 | 103.5 |  |
|  |  | Residuals | 6.5 | -6.5 |  |
|  | UTI present (1) | Counts | 4 | 39 | 43 |
|  |  | Expected | 10.5 | 32.5 |  |
|  |  | Residuals | -6.5 | 6.5 |  |
|  | **Total** | | **44** | **136** | **180** |

TP = 39, FP = 96

TP: True Positives; FP: False Positives. Model 1: guideline-based model inclusion of only specific urinary symptoms and positive leukocyte or nitrite dipstick results.Model 2: as per current guideline or ≥1 non-specific symptoms with positive leukocyte or nitrite dipstick results (extended model)
